# Urinary Nucleosides as Biomarkers of Breast, Colon, Lung, and Gastric Cancer in Taiwanese

**DOI:** 10.1371/journal.pone.0081701

**Published:** 2013-12-19

**Authors:** Wei-Yi Hsu, Chao-Jung Chen, Yu-Chuen Huang, Fuu-Jen Tsai, Long-Bin Jeng, Chien-Chen Lai

**Affiliations:** 1 Department of Medical Research, China Medical University Hospital, Taichung, Taiwan; 2 China Medical University, Taichung, Taiwan; 3 Graduate Institute of Integrated Medicine, China Medical University, Taichung, Taiwan; 4 School of Chinese Medicine, China Medical University, Taichung, Taiwan; 5 Department of Health and Nutrition Biotechnology, Asia University, Taichung, Taiwan; 6 Department of Surgery, China Medical University Hospital, Taichung, Taiwan; 7 Institute of Molecular Biology, National Chung Hsing University, Taichung, Taiwan; 8 Graduate Institute of Chinese Medical Science, China Medical University, Taichung, Taiwan; Health Canada and University of Ottawa, Canada

## Abstract

Urinary nucleosides are associated with many types of cancer. In this study, six targeted urinary nucleosides, namely adenosine, cytidine, 3-methylcytidine, 1-methyladenosine, inosine, and 2-deoxyguanosine, were chosen to evaluate their role as biomarkers of four different types of cancer: lung cancer, gastric cancer, colon cancer, and breast cancer. Urine samples were purified using solid-phase extraction (SPE) and then analyzed using high-performance liquid chromatography-tandem mass spectrometry (HPLC-MS/MS). The Mann-Whitney *U* test and Principal Component Analysis (PCA) were used to compare differences in urinary nucleosides between patients with one of four types of cancer and healthy controls. The diagnostic sensitivity of single nucleosides for different types of cancer ranged from 14% to 69%. In contrast, the diagnostic sensitivity of a set of six nucleosides ranged from 37% to 69%. The false-positive identification rate associated with the set of six nucleosides in urine was less than 2% compared with that of less than 5% for a single nucleoside. Furthermore, combining the set of six urinary nucleosides with carcinoembryonic antigen improved the diagnostic sensitivity for colon cancer. In summary, the study show that a set of six targeted nucleosides is a good diagnostic marker for breast and colon cancers but not for lung and gastric cancers.

## Introduction

Davis et al. [1] were the first researchers to compare the concentration and ratio of urinary nucleosides between healthy individuals and patients with cancer. The nucleosides were isolated with a boronate affinity column and then separated using reversed-phase high-performance liquid chromatographic (HPLC)–ultraviolet (UV) methods.

Since then, different technologies have been applied to identify and quantify target nucleosides or novel modified-nucleosides [2]. Sample preparation normally involves an extraction procedure to obtain well-purified and concentrated nucleosides from complex matrices in urine prior to instrumental analysis. Common pretreatment methods include solid phase extraction (SPE) with phenylboronic acid (PBA) as an extraction bed or gel for purification of vicinal hydroxyl groups involved in the structure of nucleosides [3], [4], cation-exchange extraction for purification of positively charged nitrogen in nucleosides and deoxynucleosides [5], and C18 extraction for purification of hydrophobic moieties in nucleosides and deoxynucleosides [6], [7]. Separation of urinary nucleosides is often carried out by chromatographic methods such as HPLC or Ultra Performance Liquid Chromatography (UPLC) [3], [5], [7], [8] or electromigration techniques such as capillary electrophoresis [9], [10]. The final stage involves the use of different types of detectors such as UV [10] or mass spectrometry (MS) [3], [8]. It is well known that MS detection provides more molecular weight information (especially when tandem MS is used) and provides higher sensitivity and specificity than UV detection. Nowadays, separation techniques combined with MS are the most commonly used techniques for identification of novel nucleosides [7] and quantification of targeted nucleosides in urine.

Sove et al. [11] demonstrated that 8-hydroxyguanosine was a good biomarker of breast cancer. Cho et al. [12] evaluated the accuracy and specificity of fourteen nucleosides for detecting breast cancer before and after tumor removal. In addition, Seidel et al. [13] reported that eighteen nucleosides could be used to predict at least nine different kinds of cancer. Furthermore, Woo et al. [6] found that a set of fourteen nucleosides could be used as a biomarker of breast, ovarian, and cervical cancer, and Szymanska et al. [10] reported that a set of nineteen nucleosides was predictive of bladder, prostate, kidney, and testicular cancer. In the present study, we investigated whether a set of six urinary nucleosides could serve as a universal biomarker of four different types of cancer in Taiwanese.

## Materials and Methods

### Chemicals and standard solutions

The nucleosides examined in this study were purchased from Sigma (St Louis, MO):

cytidine, 3-methylcytidine, 1-methyladenosine, adenosine, inosine, 2′-deoxyguanosine, and tubercidin (internal standard, ISTD). HPLC-grade methanol and acetonitrile were purchased from LAB-SCAN Analytical Science (Labscan Ltd. Dublin, Ireland). Deionized water (Milli-Q water system, Millipore Inc., Bedford, MA) was used in the preparation of the samples and buffer solutions.

Stock solutions of the six nucleoside standards and the internal standard were prepared at concentrations ranging from 100–1000 µg/ml in methanol and kept in the dark at −20°C until used.

### Instrumentation

The HPLC-ESI-MS/MS system consisted of a Surveyor™ HPLC system coupled with a Finnigan LCQ DECA XP^PLUS^ quadrupole ion trap mass spectrometer. The separation of analytes was performed on a 3 µm C18 column (Atlantis@dC18, 2.1 mm i.d×100 mm, Waters). A guard column (Atlantis@dC18, 2.1 mm i.d×20 mm, Waters) was used to prolong the life of the HPLC column. The LCQ DECA XP^PLUS^ MS system was equipped with a pneumatically-assisted electrospray ionization source and operated in positive ion mode by applying a voltage of 4 kV to the ESI needle. The temperature of the heated capillary in the ESI source was set at 295°C. The number of ions stored in the trap was regulated by automatic gain control, which was set at 2×10^7^ in selective reaction monitoring (SRM) mode. The flow rate of the sheath gas (nitrogen) was set at 30 (arbitrary units).

Helium was used as the damping gas at a pressure of 10^−3^ Torr. Voltages across the capillary and the octapole lenses were tuned by an automated procedure to maximize signals of the ion of interest. In the MS/MS analysis, typical values for the relative collision energy (peak-to-peak amplitude of the resonance excitation) ranged from 0.4 to 0.8 eV. For quantitative experiments in SRM mode, the maximum ion collection time was 0.15 s for each step and each spectrum was scanned 3 times.

### Collection of specimens

Urine samples from 149 subjects, including 26 with colon cancer, 36 with breast cancer, 27 with lung cancer, 15 with gastric cancer, and 45 healthy controls were collected at the China Medical University Hospital, Taichung, Taiwan. Patients with cancer had various stages of malignant disease and underwent different therapies (surgery, chemotherapy, radiotheraphy). Cancer was graded using the TNM (Tumor-Node-Metastasis) classification system in all patients. The details of the patients with gastric or lung cancer are described in Table 1. Clinical characteristics of the patients with colon or breast cancer are described in our previous reports [5], [14]. Urine samples were acidified using 2N HCl (adjusted to 0.01N HCl) and stored at −80°C in the dark until analysis. The study was approved by the ethical committee of the China Medical University Hospital. All participants have provided their written consent to participate in this study. The ethical committee of the China Medical University Hospital have approved this consent procedure

**Table 1:** Clinical characteristic of colon and lung cancer patients.

Patient no.	Sex	Age	TMN Stage	Cell type
1. Gastric cancer patients				
G2	M	61	T3 N0 M0 Stage II	adenocarcinoma
G4	M	69	T3 N2 M0 Stage IIIB	adenocarcinoma
G6	M	78	T3 N1 M0 Stage IIIA	adenocarcinoma
G7	M	65	Tx Nx M1 Stage IV	adenocarcinoma
G8	M	73	T3 N2 M0 Stage IIIB	adenocarcinoma
G9	F	56	T4a Nx M1 Stage IV	adenocarcinoma
G10	F	82	T4b Nx Mx Stage x	adenocarcinoma
G11	M	65	T2a N2 M0 Stage IIIA	adenocarcinoma
G14	M	53	T3 N0 M0 Stage II	adenocarcinoma
G16	M	59	T2 N1 M0 Stage II	adenocarcinoma
G17	M	50	T1 N0 M0 Stage IA	adenocarcinoma
G20	M	82	T3 N0 M0 Stage II	adenocarcinoma
G21	F	78	T1 N0 M0 Stage IA	adenocarcinoma
G27	M	54	T4 N2 M0 Stage IV	adenocarcinoma
G28	M	81	Tx Nx M1 Stage IV	adenocarcinoma
2. Lung cancer patients				
L1	M	74	T4 N0 Mx Stage IIIB	squamous cell
L2	M	73	T3 N2 M1 Stage IV	squamous cell
L3	M	71	limited disease	small cell
L4	F	56	T2 N0 M0 Stage IB	adenocarcinoma
L5	M	56	T4 N2 Mx Stage IIIB	adenocarcinoma
L6	F	58	T2 N3 M1 Stage IV	adenocarcinoma
L7	M	68	T4 N3 Mx Stage IIIB	adenocarcinoma
L8	F	61	T2 Nx M1 Stage IV	adenocarcinoma
L9	M	72	T4 N3 Mx Stage IIIB	squamous cell
L10	F	55	T4 N2 M1 Stage IV	small cell
L12	M	49	T4 N1 M0 Stage IIIB	squamous cell
L13	F	43	T1 N2 M1 Stage IV	adenocarcinoma
L14	M	66	T3 N2 M1 Stage IV	adenocarcinoma
L15	F	54	T2 N1 M0 Stage IV	squamous cell
L16	M	64	T2 Nx M1 Stage IV	adenocarcinoma
L17	M	60	T1 N3 M1 Stage IV	adenocarcinoma
L18	M	50	T2 N3 M1 Stage IV	adenocarcinoma
L20	F	46	T3 N2 M1 Stage IV	adeno-squamous cell
L21	M	81	T4 N2 M0 Stage IIIB	squamous cell
L22	M	68	T4 N3 Mx Stage IIIB	squamous cell
L23	M	65	T2 N2 M1 Stage IV	adenocarcinoma
L24	F	67	T4 Nx M1 Stage IV	adenocarcinoma
L25	F	66	Stage IV	adeno-squamous cell
L26	F	64	T2 N0 M0 Stage IIIB	adenocarcinoma
L29	M	70	T2 N3 M1 Stage IV	adenocarcinoma
L30	F	42	T2 N3 M1 Stage IV	adenocarcinoma
L31	M	42	T2 N2 M1 Stage IV	squamous cell

### Sample preparation and HPLC-MS/MS analysis

Concentrations of nucleosides in urine were quantified by SPE with HPLC-MS/MS as reported previously [5], [14]. Briefly, 1 ml of urine was added to 100 µl of internal standard (2 ug/mL) and subsequently passed through a 96-well cation-exchange cartridge (Oasis@ MCX column, Waters) to extract urinary nucleosides. The eluate from the SPE cartridge was then reconstituted in a 100 µl solution of the mobile phase (2 mmol/l aqueous ammonium acetate, pH 5.0). The concentrated urine (2 µl) was then injected into the HPLC system for analysis. The mobile phases of HPLC system were (A) 2 mmol/l aqueous ammonium acetate (pH 5.0) and (B) 50% methanolic 2 mmol/l ammonium acetate. The flow rate was 0.2 ml/min. The gradient conditions were as follows: isocratic elution (95% A) for 5 min, followed by a 2 min gradient to 20% B, then a gradient to 30% B in 3 min, then the final gradient to 40% B in 10 min. Typically, the analysis lasted 20 min and an additional 15 min was required to re-equilibrate the column. Front eluent (retention time <3 min) was diverted to waste rather than the mass spectrometer to void the ionic and polar components, thus minimizing contamination of the electrospray source.

The MS/MS experiment with SRM transitions was set for the quantification of nucleosides as follows: cytidine, m/z 244→112; 3-methylcytidine, m/z258→126; 1-methyladenosine, m/z 282→150; adenosine, m/z 268→136; inosine, m/z 269→137; 2′-deoxyguanosine, m/z 268→152 and tubercidin, m/z 267→135. To compensate for variations in urine concentration, all nucleoside concentrations were indexed against creatinine and expressed as nmol nucleoside/µmol creatinine. The urinary creatinine levels were determined by a modified Jaffe method whose principle is the reaction between creatinine and picric acid using colorimetric detection. [15]

### Statistical analyses

Alterations between the nucleosides obtained for different groups (total cancer patients versus normal controls and individual cancer patients versus normal controls) were evaluated by the Mann-Whitney *U* test. Breast cancer patients were compared with healthy females. Principal Component Analysis (PCA) was used to recognize patterns and was also applied to check the dataset structure and assess the variability of the profiles belonging to groups of total cancer patients versus normal controls and individual cancer patients versus normal controls. The relationships between urinary nucleosides levels and tumor stages of individual cancer groups were analyzed by Spearman's correlation test. All statistical analyses were performed with the statistical package SPSS for Windows (Version 17, SPSS, Chicago, Il, USA).

## Results and Discussion

### HPLC-ESI-MS/MS

**Figure 1:**
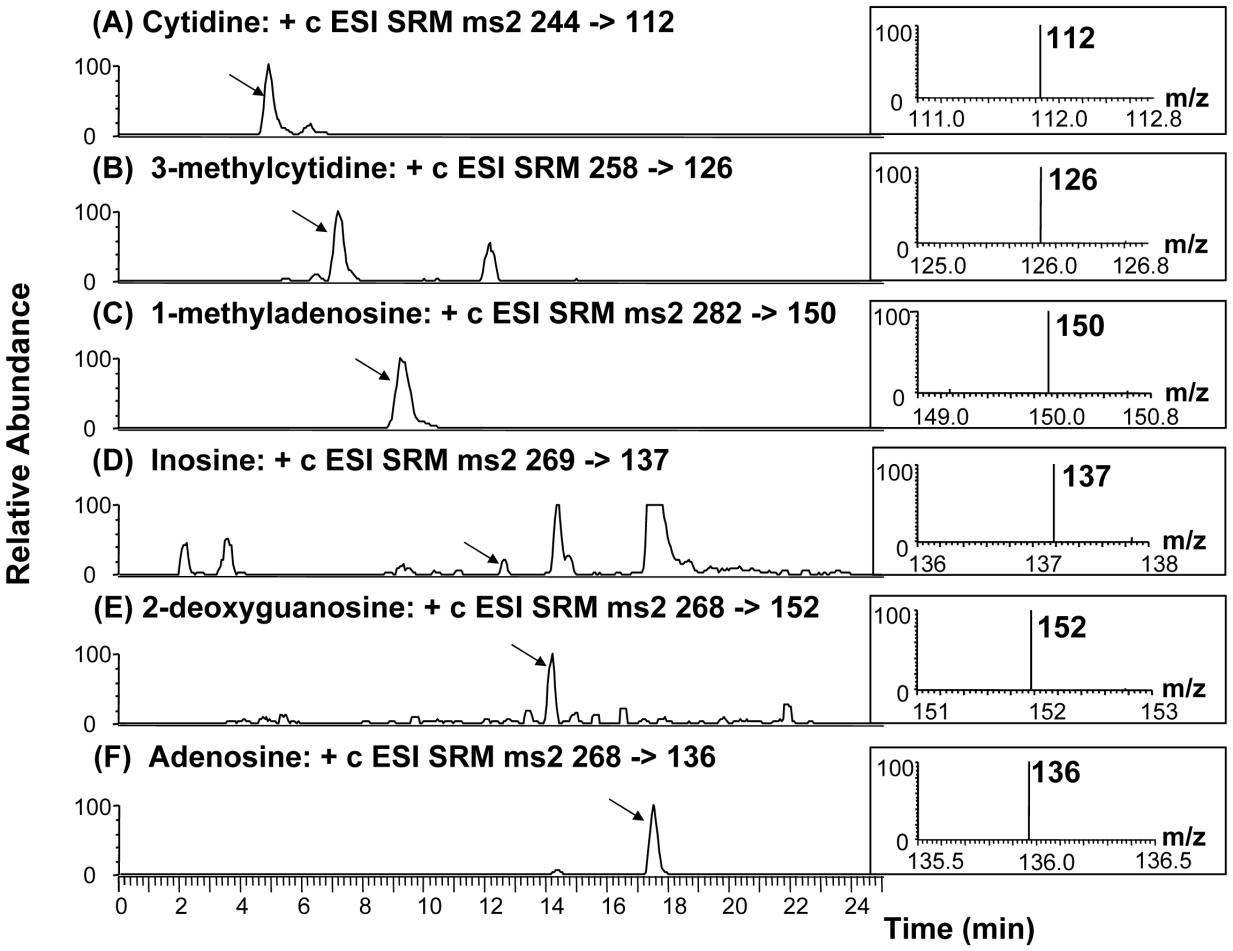
HPLC spectra of six nucleosides in pooled urine from cancer patients. Insets show the SRM transition mass spectra of each nucleoside.

In this study, an HPLC- ESI-MS/MS method was used to identify and quantify urinary nucleosides. Chromatographic retention time and specific mass information (both parent ion and daughter ion) of nucleosides were used to confirm with those of their relative standards. MS/MS (SRM transition was used in this study) detected molecular mass and structural information, allowing greater accuracy at identifying compounds than traditional UV detection. In other words, the coexisting eluents from HPLC were separated by MS detection if they had different molecular mass patterns. As seen in Fig. 1, adequate chromatographic separation of the six urinary nucleosides for the pooled cancer patients was achieved. The insets show the SRM transition spectra of each nucleoside. The parent ion, protonated nucleoside [M+H]^+^ was the most abundant ion. After collision-induced dissociation, the daughter ion, protonated base moiety [BH_2_]^+^, was analyzed with mass spectrometry. This protonated base moiety was a product of the breakdown of the glycosidic bond from the loss of a ribose moiety [MH-132]^+^ (for cytidine, 3-methylcytidine, 1-methyladenosine, adenosine, inosine, tubercidine) or 2′-deoxyribose moiety [MH-116]^+^ (for 2′-deoxyguanosine). The methods used to determine accuracy, precision, linearity, and recovery were similar to those described previously [5], [14].

### Levels of urinary nucleosides

Concentrations of the six nucleosides in urine from 149 subjects were quantified by the HPLC- MS/MS method as described above. The concentrations were normalized against urinary creatinine and expressed as nmol nucleoside/µmol creatinine. The results were analyzed by the Mann-Whitney *U* test to compare differences in level of each nucleoside between different groups. As shown in Table 2, average values, standard deviation, median values and range of nucleoside levels were elevated in all cancer patients in comparison to total normal controls (about 1∼2 fold change). Significant differences (*p*<0.01) were found for five of the six nucleosides, namely cytidine, 3-methylcytidine, 1-methyladenosine, adenosine and inosine between the two groups. We also assessed the different nucleosides levels between female and male normal controls (Table 3). Higher levels of the six nucleosides were expressed in female in comparison to male normal controls. Four nucleosides, namely cytidine, 1-methyladenosine, adenosine and inosine were significantly different between the two groups. Similar results were observed in Struck's study [3].

**Table 2:** Variation of individual nucleosides levels in the urine samples from normal controls and cancer patients.

	Normal controls (n = 45)			All cancer patients (n = 104)				
Nucleosides	Ave±SD	Median	Range	Ave±SD	Median	Range	*p*-Value	Fold change
Cytidine	1.25±0.83	0.96	0.28–4.17	2.29±1.78	1.90	0.14–8.8	**0.00**	1.9
3-methylcytidine	0.81±0.27	0.76	0.28–1.38	1.19±0.8	1.07	0.22–5.88	**0.01**	1.5
1-methyladenosine	6.47±2.47	6.59	2.47–13.14	9.16±5.19	8.42	1.22–26.28	**0.00**	1.4
2-deoxyguanosine	0.15±0.13	0.09	0.03–0.55	0.22±0.24	0.13	0.02–1.18	0.41	1.7
Adenosine	2.07±0.87	1.8	0.75–4.11	3.88±2.62	3.31	0.3–15.23	**0.00**	1.9
Inosine	0.3±0.32	0.21	0.03–1.88	0.64±0.77	0.35	0.04–5.02	**0.00**	2.0

Fold change calculated by average.

*p*-Value: Mann-Whitney *U* test.

**Table 3:** Variation of individual nucleosides levels in the urine samples from female and male normal controls.

	Normal controls						
	Female (n = 25)			Mmale (n = 20)			
Nucleosides	Ave±SD	Median	Range	Ave±SD	Median	Range	*p*-Value
Cytidine	1.54±0.92	1.31	0.28–4.17	0.88±0.52	0.72	0.32–2.49	**0.00**
3-methylcytidine	0.86±0.27	0.81	0.5–1.38	0.75±0.27	0.70	0.28–1.34	0.21
1-methyladenosine	7.41±2.59	7.34	2.93–13.14	5.28±1.73	5.37	2.47–8.12	**0.01**
2-deoxyguanosine	0.19±0.17	0.15	0.03–0.55	0.11±0.07	0.08	0.03–0.23	0.39
Adenosine	2.43±0.9	2.55	0.92–4.11	1.61±0.57	1.50	0.75–3.18	**0.00**
Inosine	0.32±0.22	0.28	0.06–0.9	0.27±0.42	0.16	0.03–1.88	**0.02**

*p*-Value: Mann-Whitney *U* test.

Next we evaluated the variation in urinary nucleosides between different types of cancer and normal controls. The group of breast cancer patients was compared with female normal controls only. We found that different sets of nucleosides were significantly associated with different types of cancer (Table 4). For example, cytidine, 3-methylcytidine, and inosine were significantly elevated in breast cancer patients; and cytidine, 1-methyladenosine, and adenosine were significantly elevated in colon cancer patients. Adenosine was the only nucleoside that was significantly elevated in urine from patients with lung cancer. However, there was no nucleoside significantly elevated in urine from patients with gastric cancer (*p*>0.05). Subsequently, cross-comparison between different cancer groups was further examined. Data (Table S1) showed that adenosine level in colon cancer group was significantly different from lung, gastric and breast cancer groups. Nucleosides levels were no significantly different between gastric and lung cancer. However, urinary nucleosides levels in breast cancer, a hormone-dependent cancer were more variable when comparing with other non hormone-dependent cancers (female only). The variable pattern of nucleosides in patients with various kinds of cancer may be due to the heterogeneity of different cancers. Studies have shown that nucleosides in urine are commonly elevated in cancer patients because of the increased tRNA methyltransferase activity or tRNA turn-over rate and that the elevated level of nucleosides excreted in urine is associated with kinetic growth parameters of different cancers.[16], [17] Besides, we also examined the relationship of urinary nucleosides levels from individual cancers with each tumor stages by Spearman's rho correlation test. Data (Table S-2) showed that adenosine levels were moderate to good correlate to tumor stages in lung cancer patients (Spearman's rho  = 0.59, *p*<0.05). However, no other significant correlation was observed in other three kinds of cancer groups.

**Table 4:** Variation of individual nucleosides levels in the urine samples from breast, lung, gastric and colon cancer patients.

	Breast cancer (n = 36)					Lung cancer (n = 27)				
Nucleosides	Ave±SD	Median	Range	*p*-Value*	Fold change*	Ave±SD	Median	Range	*p*-Value**	Fold change**
Cytidine	3.01±2.18	2.49	0.14–8.8	**0.00**	1.95	1.59±1.15	1.43	0.28–5.99	0.17	1.27
3-methylcytidine	1.57±0.74	1.56	0.3–3.18	**0.00**	1.81	0.97±0.38	0.9	0.45–1.66	0.11	1.20
1-methyladenosine	9.37±5.06	9.09	1.22–19.12	0.21	1.26	7.49±2.76	7.45	3.42–14.04	0.16	1.16
2-deoxyguanosine	0.24±0.18	0.16	0.08–0.75	0.33	1.31	0.32±0.34	0.15	0.03–1.18	0.11	2.13
Adenosine	3.34±1.84	3.20	0.3–7.38	0.13	1.37	2.84±1.39	2.62	1.05–5.68	**0.02**	1.38
Inosine	0.94±0.74	0.82	0.11–3.36	**0.00**	2.90	0.36±0.3	0.29	0.04–1.06	0.38	1.20

	Gastric cancer (n = 15)					Colon cancer (n = 26)				
Nucleosides	Ave±SD	Median	Range	*p*-Value**	Fold change**	Ave±SD	Median	Range	*p*-Value**	Fold change**
Cytidine	2.18±1.79	1.89	0.29–6.69	0.07	1.75	2.08±1.35	1.80	0.57–5.77	**0.00**	1.67
3-methylcytidine	1.26±1.4	1.00	0.3–5.88	0.40	1.54	0.86±0.55	0.64	0.22–2.56	0.55	1.05
1-methyladenosine	10.77±7.65	9.14	2.6–26.28	0.05	1.67	9.71±5.42	8.80	1.8–24.17	**0.01**	1.50
2-deoxyguanosine	0.12±0.11	0.07	0.04–0.35	0.45	0.78	0.15±0.19	0.08	0.02–0.81	0.45	1.00
Adenosine	3.41±2.51	2.74	0.57–9.2	0.15	1.65	5.97±3.4	5.37	0.76–15.23	**0.00**	2.89
Inosine	0.98±1.43	0.37	0.06–5.02	0.10	3.28	0.3±0.33	0.19	0.06–1.21	0.78	1.00

* Compared with "female normal controls".

** Compared with "total normal controls".

Fold change calculated by average.

*p*-Value: Mann-Whitney *U* test.

**Figure 2:**
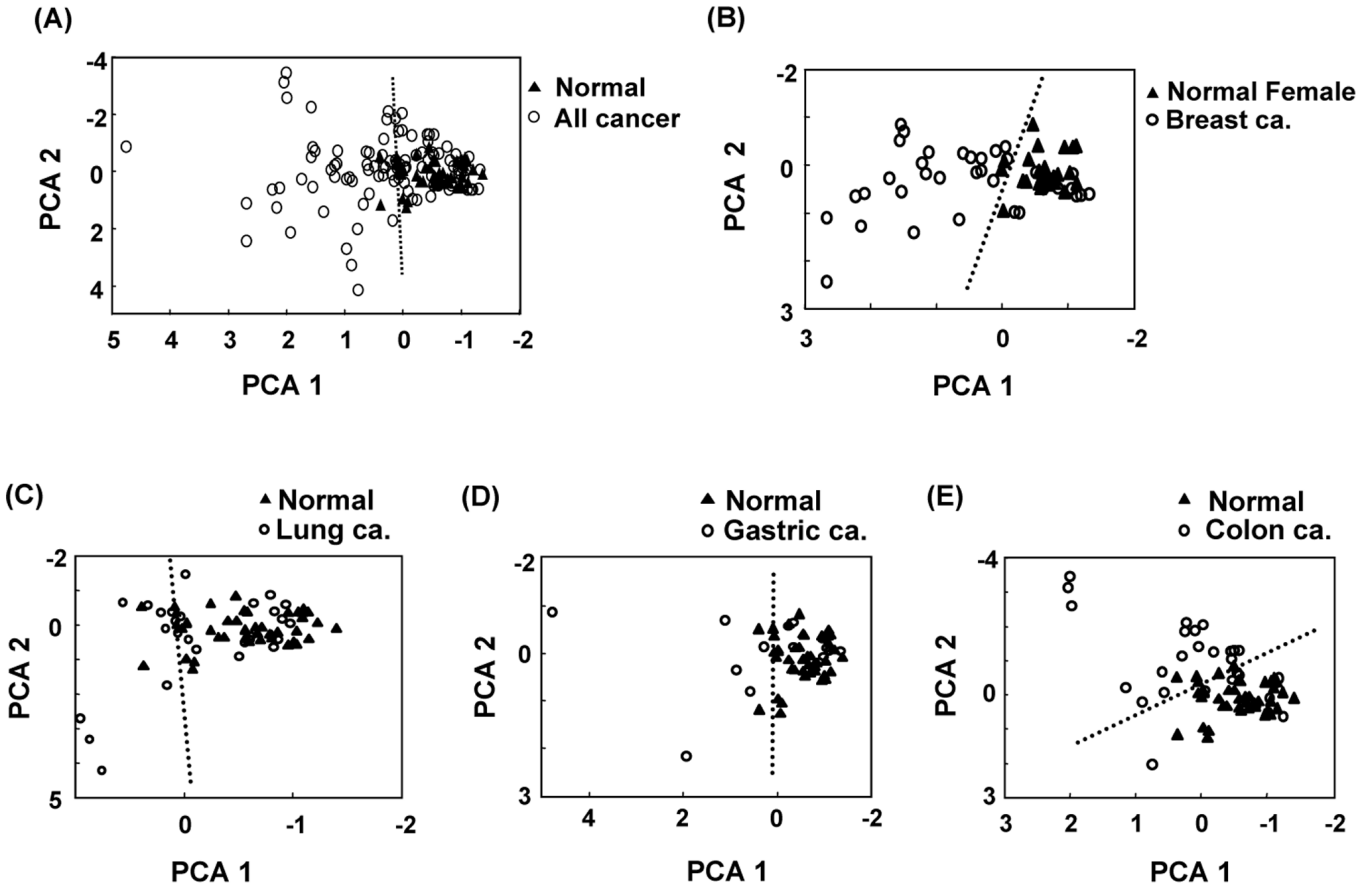
Results of principal component analysis showing the distribution of cancers and normal controls. (A) All cancer; (B) Breast cancer, (C) Lung cancer; (D) Gastric cancer and (E) Colon cancer. Black triangles mark normal controls, while the white circle marks each cancer group.

The profiles of nucleosides in urine from cancer patients and normal controls were explored by PCA to assess the strength of the relationship between nucleoside profiles and cancer. Additionally, the extracted PCA factors were used to examine the ability to discriminate between cancer patients and normal controls. PCA showed that the total variance of patients and controls explained by the first PCA (PCA 1) was 51% whereas the second PCA (PCA 2) explained nearly 17% of the variance. PCA 1 was affected mostly by metabolites 1-methyladenosine (0.87) and 3-methylcytidine (0.84) and PCA 2 was influenced mostly by 2-deoxyguanosine (0.69). The PCA 1/PCA 2 score plots revealed that the samples from normal controls were more homogeneous than those from patients with cancer (Fig. 2A). The result was consistent with previous reports that PCA analysis of cancer patients were more dispersed, which were associated with their high diversity in tumor stage and cancer type.[3] Furthermore, as seen in Fig. 2B–E, we obtained a clear separation between normal controls and individual cancer groups.

### Comparison of diagnostic sensitivity of a single nucleoside and a set of six nucleosides

**Figure 3:**
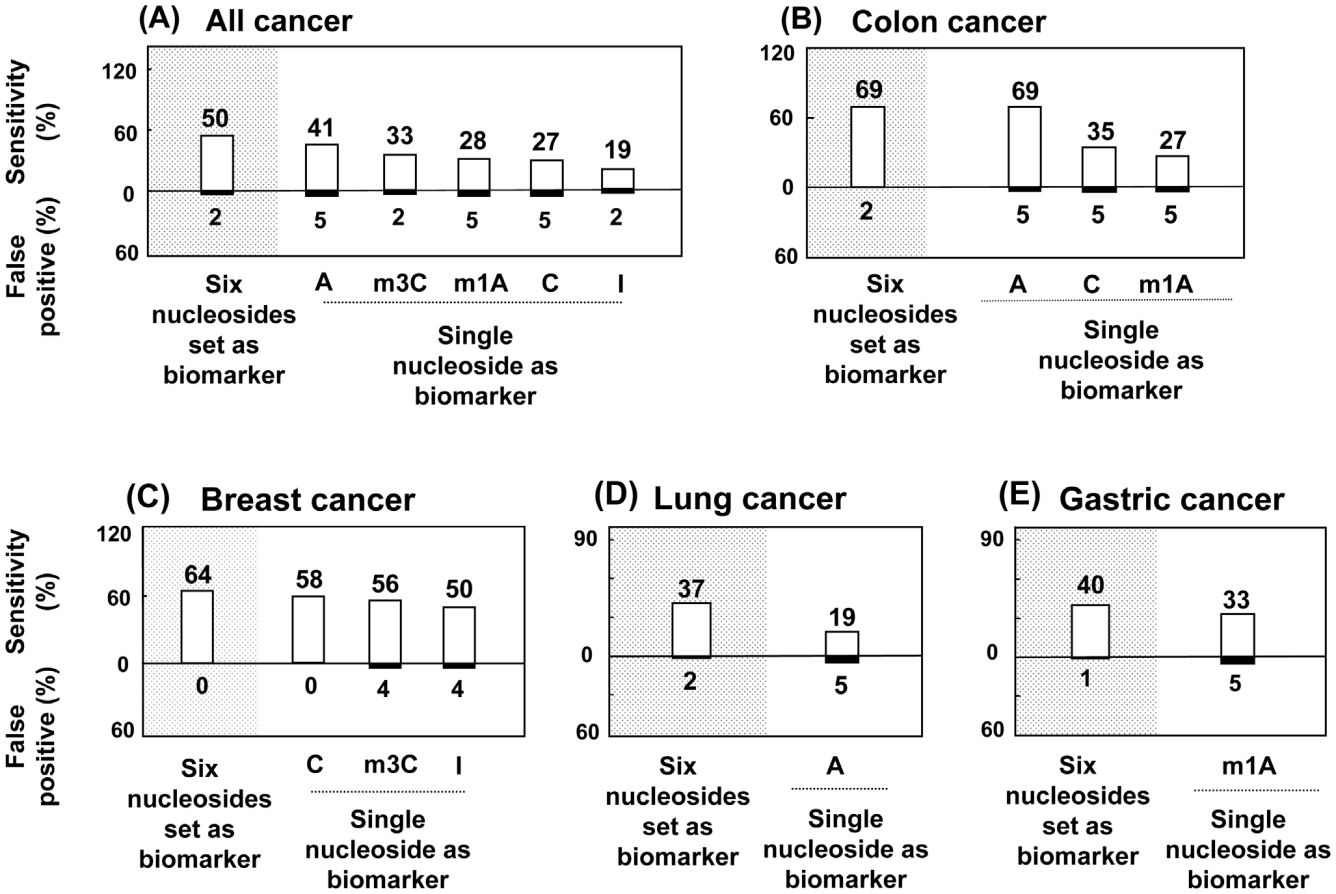
Diagnostic sensitivity and false-positive identify for cancers provided by a single nucleoside and the set of six nucleosides. (A) All cancer; (B) Colon cancer, (C) Breast cancer; (D) Lung cancer and (E) Gastric cancer.

On the basis of average values obtained from the normal controls, a cut-off value was defined as average plus 2-fold standard deviation for each nucleoside to decrease the false-positive identification rate of normal controls to less than 5%. This was also related to a specificity greater than 95%, which was defined as correctly classified normal controls in previous reports [3], [18]. The percentage of each cancer group with a urinary nucleoside (significantly variation, p<0.01) level above the cut-off value was calculated and used to define diagnostic sensitivity. In the group of gastric cancer, 1-methyladenosine (*p* = 0.052) was chosen as candidate nucleoside marker. Breast cancer patients were compared with female normal controls. A set of six nucleosides was also defined as a diagnostic marker by the result of PCA analysis, and a dotted line was used as a boundary to separate the normal control group from the cancer groups. The percentage of cancer patients on one side of the boundary was calculated and used to define diagnostic sensitivity. Also, the false-positive identification rate of normal controls was calculated as the percentage of normal controls on same side of the boundary. The specific line across the PCA scatter plot was selected to obtain the maximal value of sensitivity and minimal value of false-positive. Fig. 3 shows the diagnostic sensitivity (white box) of the methods for detecting each type of cancer and the false-positive (black box) rate in the normal controls. Also, we compared the diagnostic strengths provided by a single nucleoside with those provided by the set of six nucleosides. In the “all cancer” group, the diagnostic sensitivities ranged from 19% to 41% for the single nucleoside. The diagnostic sensitivity of the set of six nucleosides was, however, approximately 50% (50 of 104 patients). In the colon cancer group, the diagnostic sensitivity was 69% (18 of 26 patients) for the single nucleoside (adenosine) and for the set of six nucleosides. The false-positive identification rate decreased from 5% to 2% when the set of six nucleosides was used. In the other three groups, the set of six nucleosides provided a higher diagnostic sensitivity of 64% (23 of 36 patients) for breast cancer; 37% (10 of 27 patients) for lung cancer and 40% (6 of 15) for gastric cancer than single nucleoside marker. The false identification rate decreased from 5% to 2% (for lung cancer) and to 1% (for gastric cancer) when calculated using the set of six nucleosides.

**Figure 4:**
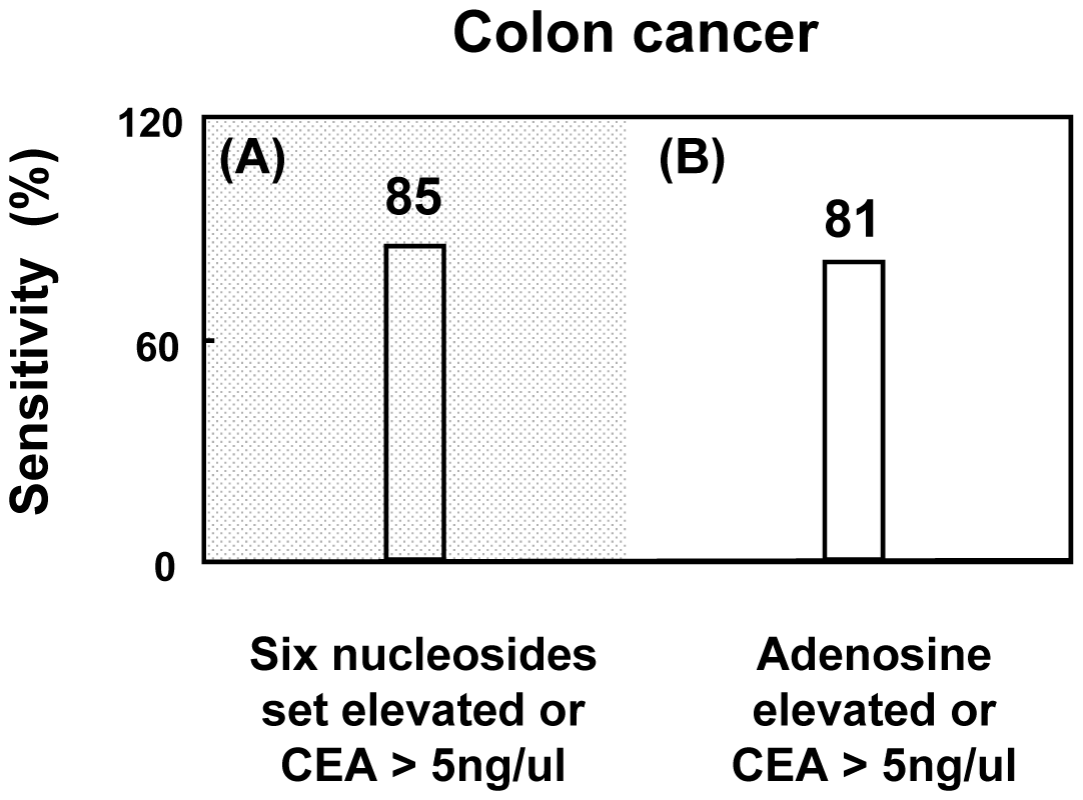
Diagnostic sensitivity for colon cancers provided by combination of serum carcinoembryonic antigen (CEA>5 ng/ul) with urinary (A) adenosine or (B) set of six nucleosides.

Next, we combined the urinary nucleoside values with the value of serum carcinoembryonic antigen (CEA) from patients with colon cancer to improve the diagnostic sensitivity (Fig. 4). Serum CEA is a rotine diagnostic marker for colon cancer in our hospital. Of the 26 colon cancer patients, cancer can not detected using the set of six urinary nucleosides and 4 patients had higher serum CEA levels (cut-off value of 5 ng/ul). Therefore, the diagnostic sensitivity of the method at detecting colon cancer increased from 68 to 85% (4+18 of 26 patients). In the same way, combination of the urinary nucleoside adenosine and serum CEA improved the diagnostic sensitivity of the method at detecting colon cancer from 68% to 81% (3+18 of 26 patients). The results show that integration of urinary nucleosides with rotine markers elevates the diagnostic sensitivity of detecting cancer.

The set of six nucleosides was a good diagnostic marker for breast and colon cancer (diagnostic sensitivity >60%, false-positive identification <2%), but not for lung and gastric cancer (diagnostic sensitivity <40%). Few studies have evaluated the relationship between urinary nucleosides and lung or gastric cancer. McEntire et al [18] reported that serum nucleosides, other than urinary nucleosides, had a sensitivity of 84% and a specificity of 79% for detecting lung cancer. They reported that serum nucleosides may be subjected to fewer structural alternations than urinary nucleosides and may account for higher serum levels of some nucleosides.

## Conclusion

We used a well-established SPE-HPLC-MS/MS method to quantify urinary nucleosides. The MS-based method was analytically sensitive and specific and was capable of analyzing multiple nucleosides in a single run of HPLC. Our results show that a set of nucleosides is a good diagnostic marker of cancer and that diagnostic sensitivity can be improved by comparing urinary nucleoside levels with those of well-known serum biomarkers, like serum CEA. In this study, the set of six nucleosides was not sensitive at detecting lung and gastric cancer. It is, therefore, necessary to consider different nucleosides for different kinds of cancer.

## Supporting Information

Table S1 **Variation of individual nucleoside levels in the urine samples between lung, colon, gastric and breast cancer patients.**
(DOC)

Table S2 **S Spearman's rho correlation coefficients between individual nucleosides levels and tumor stages (0 to IV) in each cancer patients.**
(DOC)
