# Supplementary material for: Urinary Nucleosides as Biomarkers of Breast, Colon, Lung, and Gastric Cancer in Taiwanese
Source: PLoS One. 2013 Dec 19;8(12):e81701. doi: 10.1371/journal.pone.0081701 (PMC3868621; doi:10.1371/journal.pone.0081701)
Supplement: Table S1 — Variation of individual nucleoside levels in the urine samples between lung, colon, gastric and breast cancer patients. (DOC) [file pone.0081701.s001.doc]

|  | Mann-Whitney *U* test (*p*-Value) | | |
| --- | --- | --- | --- |
| Nucleosides | Lung ca. vs. Gastric ca. | Lung ca. vs. Colon ca. | Gastric ca. vs. Colon ca. |
| Cytidine | 0.47 | 0.15 | 0.83 |
| 3-methylcytidine | 0.95 | 0.12 | 0.41 |
| 1-methyladenosine | 0.28 | 0.15 | 0.92 |
| 2'-deoxyguanosine | 0.08 | 0.05 | 0.83 |
| Adenosine | 0.79 | **0.00** | **0.01** |
| Inosine | 0.37 | 0.34 | 0.09 |
|  |  |  |  |
|  | Mann-Whitney *U* test (*p*-Value) | | |
| Nucleosides | Bresat ca. vs. Lung ca. (female) | Breast ca. vs. Gastric ca. (female) | Breast ca. vs. Colon ca. (female) |
| Cytidine | **0.03** | 0.96 | 0.26 |
| 3-methylcytidine | **0.03** | 0.65 | **0.01** |
| 1-methyladenosine | 0.67 | 0.28 | 0.55 |
| 2'-deoxyguanosine | 0.21 | 0.41 | **0.00** |
| Adenosine | 0.77 | 0.98 | **0.00** |
| Inosine | **0.01** | 0.42 | **0.00** |

Table S1.Variation of individual nucleoside levels in the urine samples between lung, colon, gastric and breast cancer patients.
