# Supplementary material for: Urinary Nucleosides as Biomarkers of Breast, Colon, Lung, and Gastric Cancer in Taiwanese
Source: PLoS One. 2013 Dec 19;8(12):e81701. doi: 10.1371/journal.pone.0081701 (PMC3868621; doi:10.1371/journal.pone.0081701)
Supplement: Table S2 — S Spearman's rho correlation coefficients between individual nucleosides levels and tumor stages (0 to IV) in each cancer patients. (DOC) [file pone.0081701.s002.doc]

|  | Tumor stage | | | |
| --- | --- | --- | --- | --- |
| Nucleosides | Breast cancer | Lung cancer | Gastric cancre | Colon cancer |
| Cytidine | 0.20 | 0.25 | -0.30 | 0.19 |
| 3-methylcytidine | 0.10 | 0.26 | -0.20 | 0.13 |
| 1-methyladenosine | 0.05 | 0.36 | -0.02 | 0.19 |
| 2'-deoxyguanosine | -0.26 | 0.13 | -0.28 | 0.19 |
| Adenosine | -0.01 | **0.59*** | -0.15 | 0.26 |
| Inosine | -0.24 | 0.17 | -0.02 | 0.25 |

*Level of significant *p*<0.05.

Table S2. Spearman's rho correlation coefficients between individual nucleosides levels and tumor stages (0 to IV) in each cancer patients.
